# Lumbar paraspinal muscles in patients with chronic non-specific low back pain and the effect of exercise: comparative and interventional study

**DOI:** 10.3389/fresc.2025.1714052

**Published:** 2025-12-11

**Authors:** Viktoria Kokosova, Daniela Vlazna, Peter Krkoska, Michaela Sladeckova, Marek Dostal, Katerina Matulova, Radim Gerstberger, Petra Ovesna, Blanka Adamova

**Affiliations:** 1 Department of Neurology, Centre for Neuromuscular Diseases (Associated National Centre in the ERN EURO-NMD), University Hospital Brno, Brno, Czechia; 2 Faculty of Medicine, Masaryk University, Brno, Czechia; 3 Department of Rehabilitation, University Hospital Brno, Brno, Czechia; 4 Department of Radiology and Nuclear Medicine, University Hospital Brno, Brno, Czechia; 5 Department of Biophysics, Faculty of Medicine, Masaryk University, Brno, Czechia; 6 Institute of Biostatistics and Analyses Ltd., Brno, Czechia

**Keywords:** magnetic resonance imaging, paraspinal muscles, muscle strength, endurance, low back pain, exercise therapy

## Abstract

**Background:**

Lumbar paraspinal muscles (LPM), which belong to lumbar extensor muscles, play a key role in stabilising the lumbar spine and maintaining spine health. Although chronic non-specific low back pain (CNLBP) represents a major health problem worldwide, there is a knowledge gap regarding the function, morphology of LPM, and the effect of regular exercise on them in patients with CNLBP. The purpose of the study was to evaluate functional and quantitative MRI (qMRI) parameters of LPM in CNLBP patients, compare them to matched healthy volunteers (HV), and assess the effect of exercise therapy on these parameters in CNLBP patients.

**Methods:**

The first part of the study was cross-sectional, observational, and comparative; the second was prospective and interventional. Based on physiological parameters, 43 matched pairs of CNLBP patients and HV were formed. All participants underwent MRI of the LPM using a 6-point Dixon gradient-echo sequence to assess qMRI parameters: fat fraction, total muscle volume, and functional muscle volume (FMV). Maximal isometric lumbar extensor muscle strength (MILEMS), lumbar extensor muscle endurance (LEME), MILEMS to LPM FMV ratio, pain, and disability were assessed. Each group's parameters were compared and effect size evaluated. The total of 29 patients entered an 18-week hybrid guided daily home-based exercise programme, which was completed by 27 CNLBP patients. The study was registered, trial registration number is ISRCTN11556477.

**Results:**

CNLBP patients had significantly lower MILEMS, LEME (both *P* < 0.001), and MILEMS to LPM FMV ratio (*P* = 0.006) compared to matched HV. These parameters, as well as pain and disability, improved significantly after exercise (each *P* < 0.001). The qMRI parameters did not differ between CNLBP patients and HV, nor did they change after exercise therapy.

**Conclusions:**

The significant LPM dysfunction and poorer muscle quality in CNLBP patients were reversible with exercise. Our study does not support the use of qMRI parameters as objective and reliable biomarkers of LPM dysfunction in a cohort of CNLBP with mild clinical impairment.

## 1 Introduction

Non-specific low back pain is an axial/non-radiating pain occurring primarily in the back without known specific pathology (e.g., infection, tumour, fracture, structural deformity, spinal stenosis, etc.) (1, 2). It is a major public health problem worldwide, affecting people of all ages, most commonly in the 40–69 age group, and more commonly in women (1, 3). Chronic non-specific low back pain (CNLBP) is characterised by pain lasting more than 12 weeks; the prevalence is approximately 23% (2, 4).

CNLBP is a multifactorial condition; complex interrelationships between biological, psychological, and social factors play a role in its development (5, 6). Among the biological factors related to CNLBP genesis, the morphology and function of the lumbar paraspinal muscles (LPM), which are part of the lumbar extensor muscles (LEM), are the most important (6). Quantitative magnetic resonance imaging (qMRI) has emerged as a rapidly advancing technique for assessing skeletal muscle structure and composition. Its increasing popularity is largely due to its non-invasive nature and its ability to provide objective biomarkers that reflect degenerative muscle changes associated with aging and various pathological conditions, such as neuromuscular disorders and low back pain (7, 8). However, the results of studies on morphological and functional parameters of LPM in CNLBP patients are inconsistent and often contradictory (9) and the understanding of their mutual correlation is limited (10, 11). The discrepancy between qMRI parameters and muscle dysfunction may be caused by the dysfunction preceding morphological muscle changes; it also varies depending on the severity and type of disease, as well as the qMRI parameters selected. When assessing the morphology and function of the LPM, it is also important to bear in mind that these are influenced by physiological variables, such as age, sex, and anthropometric parameters (10, 12–17).

Exercise has been proven to improve back muscle strength and endurance and to reduce pain and disability (18–20); exercise therapy is therefore strongly recommended in the treatment of patients with low back pain (LBP) (21). Nevertheless, it is not entirely clear whether exercise changes the morphology of LPM (18, 22, 23). The optimal exercise modalities to achieve muscular changes are not entirely clear, and the relationships between these changes and clinical improvements have not yet been fully explained (18, 24, 25). Longer-term exercise programmes, focusing initially on appropriate muscle recruitment strategies (specific sensorimotor control training), followed by strength and endurance training (with gradually increasing loads), may be necessary to achieve changes in muscles (9, 18). Recent evidence indicates a growing shift toward the implementation of hybrid and telehealth-supported rehabilitation models in the management of CLBP. Hybrid rehabilitation models, which integrate structured home-based exercise with intermittent professional oversight and remote monitoring, have gained recognition as effective and adaptable approaches (26). By enabling individualised care, these programmes fostered greater patient engagement, improved adherence, and enhanced clinical outcomes (27).

The aims of this study were: (1) to evaluate functional and qMRI parameters of the LPM in CNLBP patients and compare them to matched healthy volunteers (HV); (2) to analyse the relationship between functional and qMRI parameters of LPM in CNLBP; and (3) to assess the effect of 18-week daily hybrid guided home-based exercise therapy on functional and qMRI parameters of LPM and on patient-oriented outcomes (disability and pain) in CNLBP. The purpose of the study was to better understand the functional and morphological changes of the LPM in CNLBP patients and identify how these can be influenced by regular exercise.

## 2 Methods

This study consisted of two parts: a single-centre, cross-sectional, observational and comparative study, and a prospective and interventional study. The study protocol (agreement number 02-120220/EK dated February 12, 2020 and 22-100620/EK dated June 10, 2020) was approved by the Ethics Committee of the University Hospital Brno. All participants provided written informed consent. The study was conducted in accordance with the Declaration of Helsinki (as revised in 2013). The study was registered, trial registration number is ISRCTN11556477.

### 2.1 Participants

#### 2.1.1 Healthy volunteers

The HV were recruited between April 2020 and March 2023. Inclusion criteria were age >18 years, no history of chronic LBP, and no current LBP.

#### 2.1.2 Patients with CNLBP

Most of the patients were referred by collaborating outpatient neurologists between March 2020 and April 2023. Inclusion criteria were age between 18 and 70 years and CNLBP (pain localised in the lumbar spine area and without radiation below the knee; pain duration over 12 weeks). Discontinuation of analgesics was not required at study entry.

The exclusion criteria for both groups were: general MRI contraindications, presence of any metal material in the lumbar spine, previous lumbar spine involvement (vertebral fracture, tumour, spine infection) and/or surgery, scoliosis, the presence of lumbar spinal stenosis (Schizas classification above A4), lumbar disc herniation, comorbid conditions affecting overall mobility, pregnancy, presence of lumbosacral radicular pain in the medical history with residual clinical signs of nerve root dysfunction, and presence of myopathy.

### 2.2 Medical history, clinical examination, and functional assessment of LPM

All HV and CNLBP patients underwent a detailed medical history and clinical neurological examination to confirm eligibility for the study. Patient-oriented outcomes (pain and disability) were assessed. Pain was assessed using an 11-point Pain Numerical Rating Scale (0–10), and current pain intensity was recorded along with the average and maximum pain intensity over the previous 4 weeks; the Oswestry Disability Index (28) and the Roland-Morris Disability Questionnaire (29) were used for disability.

LPM function was assessed by measuring the maximal isometric lumbar extensor strength (MILEMS) and the lumbar extensor endurance (LEME). MILEMS (in kilograms) was examined with the subject seated in a purpose-designed chair using a handheld dynamometer MicroFET 2 (Hoggan Scientific, LLC.). Participants had five attempts, with 20 s to rest between attempts. MILEMS was calculated as the mean of the second to fifth attempts. LEME was assessed using the Biering-Sørensen test (in seconds). The functional examination methodology was described in detail in a previous article (30).

CNLBP patients were reassessed for pain, disability, and parameters of LPM function (MILEMS in sitting position and LEME) also at the end of the exercise programme.

### 2.3 MRI of lumbar spine and LPM

HV and CNLBP patients underwent MRI measurements at baseline. CNLBP patients who completed the exercise programme were also reassessed at the end.

The Philips Ingenia 3T MRI system with anterior and posterior receiving coils was used for the morphological evaluation. The examination included standard MRI sequences (turbo spin echo T2, T1, and STIR in the sagittal plane, and T2 in the axial and coronal planes). Furthermore, an axial 6-point Dixon gradient echo sequence with multi-fat-peak compensation (seven) was utilised as well as eddy current correction (labelled mDixon Quant by the Philips company) for creating water, fat, in-phase, out-phase images, and fat fraction maps with resolution 1.2*1.2*5 mm^3^.

**Figure 1:**
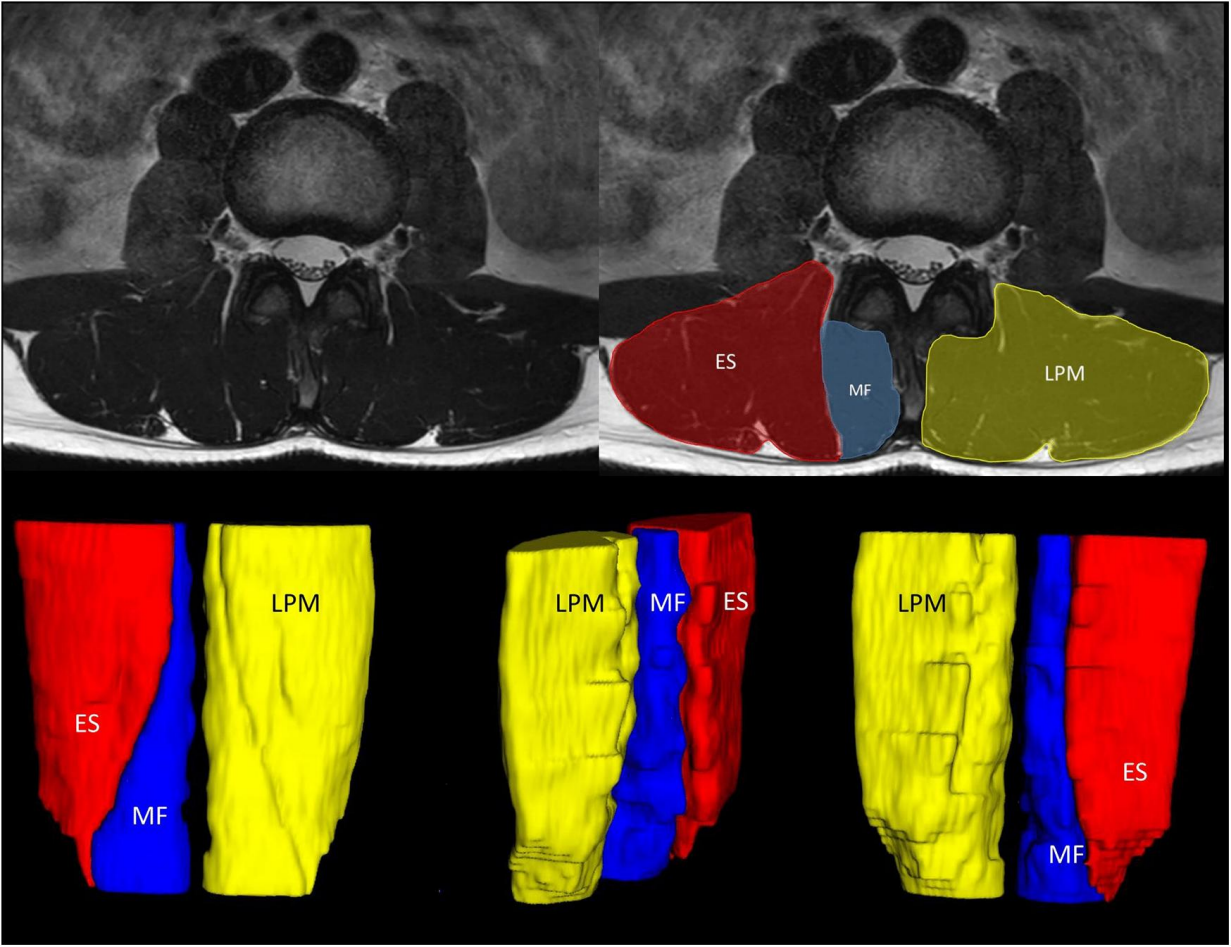
Representative figure of lumbar paraspinal muscle segmentation. MRI T2-weighted sequence of the LPM from a 33-year-old male patient with chronic non-specific low back pain. The upper figures show MRI images in the axial plane at level of the intervertebral disk L2/L3. In the figure on the right, the individual segmented muscles are delineated. The lower figure shows a 3D model illustrating segmented muscles. The LPM include the erector spinae muscle (ES) and the multifidus muscle (MF). ES, erector spinae muscle; MF, multifidus muscle; LPM, lumbar paraspinal muscles; MRI, magnetic resonance imaging; 3D, three dimensional.

The sequences covered LPM from intervertebral disc Th12/L1 to L5/S1. An experienced radiologist assessed all MRI images to exclude pathology. Manual segmentation of the bilateral multifidus muscle and erector spinae muscle was performed using ITK-SNAP software without any interpolation methods (31). The inter- and intra-rater reliability of LPM segmentation were assessed and evinced sufficient robustness (15). LPM represented multifidus and erector spinae muscle combined (Figure 1). Segmentation masks were utilised to extract muscle FF (fat fraction) and TMV (total muscle volume) of individual muscles. FF represented an average of FF in all muscle voxels bilaterally, expressed as a percentage. TMV were estimated as a sum of TMV from right and left-sided muscles. FMV (functional muscle volume) was calculated as FMV = TMV*(1-FF) (32). The methodology was described in more detail in a previous article (15). To evaluate muscle contractility, defined as the ratio of muscle strength to muscle mass, MILEMS to LPM FMV ratio was calculated (33–35).

### 2.4 Hybrid guided home-based exercise therapy

Patients with CNLBP participated in a hybrid guided home-based exercise programme, described in detail in previous works (26, 36). The comprehensive programme combined back school, respiratory training, and sensorimotor exercises engaging the lower back muscles as a part of the core system by activating and coordinating deep trunk muscles and reducing the overactivation of the superficial back muscles.

The intervention lasted 18 weeks, during which each patient attended seven scheduled physiotherapy check-ups. Three sets of exercises with progressively increasing difficulty were created. The baseline visit provided the initial set, followed by a two-week check-up for questions and verification of correct exercise performance. Another check-up visit was scheduled four weeks later to assess progress and decide on a more challenging set. Progression involved more demanding postures, increased repetitions, or longer holding times to continuously stimulate motor learning. This model of check-up consultations after two weeks and the intensity switch after another four weeks was repeated two more times.

The exercises were to be performed on two occasions every day, ideally for 15 min each time. To support adherence, each participant received illustrated booklets detailing the exercises. Compliance was monitored using telemonitoring via a mobile application, allowing investigators to promptly contact patients if adherence appeared insufficient. Patients unable or unwilling to use the app could record their exercise frequency in a paper-based diary.

### 2.5 Data analysis

Data analysis was performed in R software (v4.3.2) using two-sided tests with statistical significance set at *α* = 0.05. Given the exploratory nature of the study and the interrelatedness of the analysed parameters, we did not apply a formal multiplicity correction. The total of 43 matched pairs of CNLBP patients and HV were created utilising propensity score matching accounting for sex, age, and BMI. All statistical tests respected the data type and distribution.

To compare the basic characteristics of the two groups before matching, Welch's *t*-test and Fisher's exact test were used; after matching, the paired t-test and McNemar's chi-squared test were applied. To assess differences in qMRI and functional parameters of LPM between groups, mean paired differences and *P*-values were computed using the paired *t*-test. Next, the effect size statistic was calculated for each parameter using the effsize package (v0.8.1). Hedges' *g* expresses the standardised magnitude of the difference between two groups (37, 38), and its 95% confidence intervals were also reported to reflect the precision of the estimates. Spearman's rank correlation was used to assess relationships between functional (MILEMS and LEME) and qMRI (FF, TMV, FMV) parameters of LPM. Furthermore, to assess the association between qMRI parameters and MILEMS and LEME, univariable generalised linear regression models with gamma distribution and log link function were fitted, with MILEMS and LEME as dependent variables and FF, TMV, and FMV of LPM separately as explanatory variables. To compare the CNLBP patients before and after exercise and CNLBP patients after exercise and matched HV, mean paired differences and *P*-values were evaluated using paired t-test. Again, the effect size was reported using Hedges' *g* with corresponding 95% confidence intervals.

## 3 Results

### 3.1 Basic characteristics of groups

**Figure 2:**
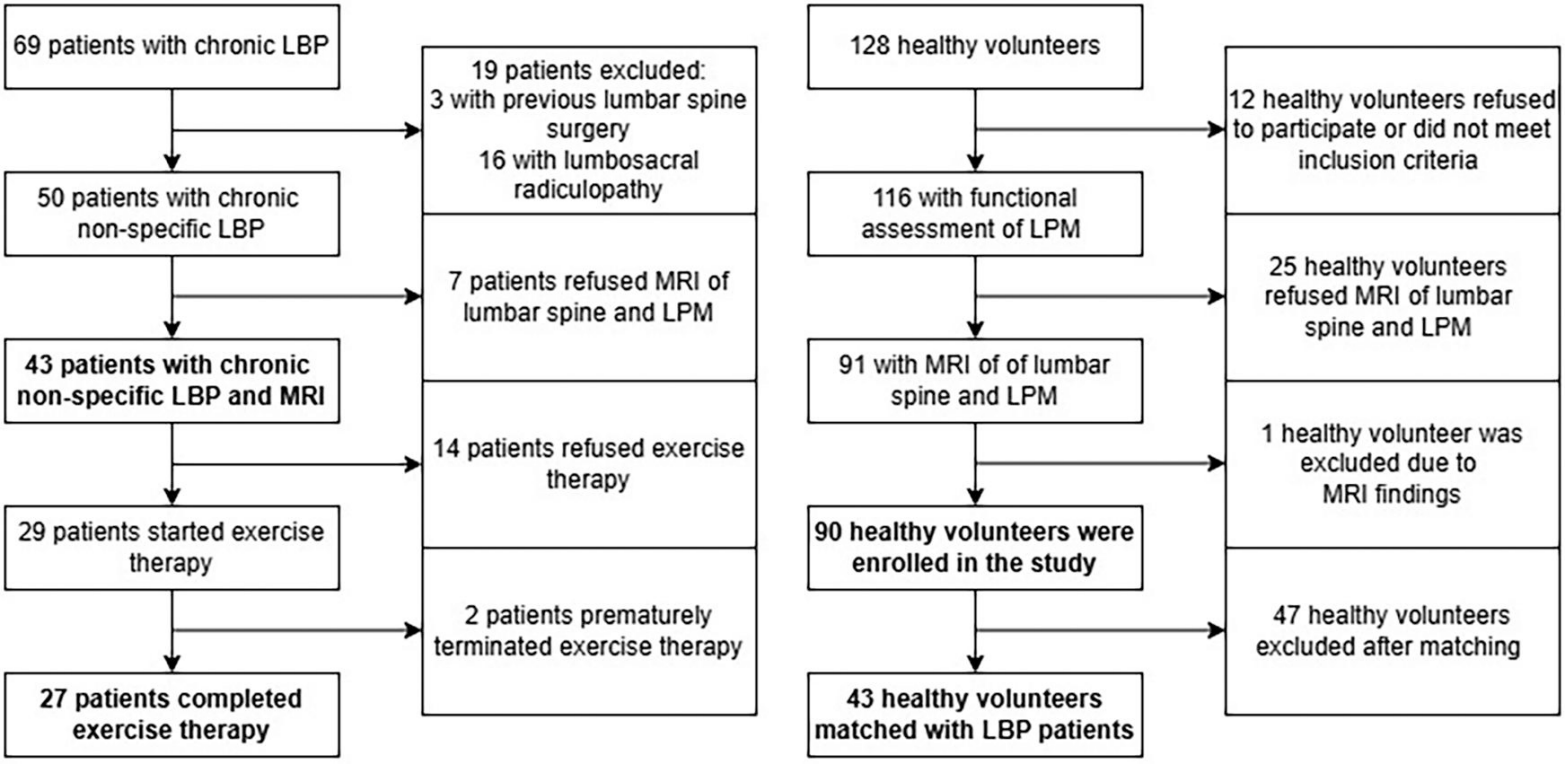
Study flowchart showing enrolment of healthy volunteers and patients with chronic non-specific low back pain.

Power analysis showed that a minimum of 47 subjects per group is required to detect statistically significant effect sizes for the primary objectives (statistical power of 0.8, significance level of 0.05). Subjects with missing data in any of considered variables were excluded from the study leaving total of 90 HV and 43 CNLBP patients for the final analysis (Figure 2). The basic characteristics of the cohorts are summarised in Table 1. The two groups differed significantly in age, weight, and BMI. After matching, we obtained a total of 43 pairs (HV-CNLBP) with same sex and similar baseline parameters (age, height, weight, BMI) (Table 1).

**Table 1:** Basic characteristics of HV and patients with CNLBP before and after matching.

Before matching			
Variable	HV *N* = 90^a^	CNLBP *N* = 43^a^	*P*-value^b^
Age (years)	39.0 (11.3)	43.7 (12.6)	**0** **.** **040**
Sex (male)	45 (50.0%)	23 (53.5%)	0.715
Height (cm)	175.2 (9.7)	174.6 (8.3)	0.680
Weight (kg)	75.7 (15.6)	81.1 (13.5)	**0** **.** **040**
BMI (kg/m^2^)	24.5 (3.8)	26.6 (4.1)	**0** **.** **005**

After matching			
Variable	HV *N* = 43^a^	CNLBP *N* = 43^a^	*P*-value^c^
Age (years)	42.8 (11.5)	43.7 (12.6)	0.621
Sex (male)	23 (53.5%)	23 (53.5%)	>0.999
Height (cm)	176.1 (9.7)	174.6 (8.3)	0.278
Weight (kg)	80.3 (13.7)	81.1 (13.5)	0.690
BMI (kg/m^2^)	25.8 (3.4)	26.6 (4.1)	0.182

BMI, body mass index; HV, healthy volunteers; CNLBP, chronic non-specific low back pain; *N*, number of subjects.

Significant *P*-values are displayed in bold.

a Mean (SD); *N* (%).

b Welch's two-sample *t*-test; Fisher's exact test.

c Paired *t*-test; McNemar's chi-squared test.

### 3.2 Functional and qMRI parameters of LPM

The functional and qMRI parameters of the LPM of the matched groups are shown in Table 2.

**Table 2:** Quantitative MRI and functional parameters of LPM in HV and CNLBP patients after matching.

Parameter		HV *N* = 43^a^	CNLBP *N* = 43^a^	Mean difference (95% CI)	*P*-value^b^	Hedges' *g* (95% CI)
Erector spinae muscle	FF (%)	10.8 (6.7)	11.5 (5.8)	0.65 (−1.65 to 2.96)	0.571	0.1 (−0.25 to 0.46)
	TMV (cm^3^)	591 (157)	565 (146)	−26.4 (−68.4 to 15.5)	0.211	−0.17 (−0.44 to 0.1)
	FMV (cm^3^)	528 (151)	501 (138)	−27.0 (−68.0 to 14.1)	0.192	−0.18 (−0.46 to 0.09)
Multifidus muscle	FF (%)	14.3 (7.9)	14.7 (8.8)	0.42 (−2.81 to 3.64)	0.796	0.05 (−0.33 to 0.42)
	TMV (cm^3^)	222 (44)	213 (56)	−8.8 (−28.8 to 11.3)	0.381	−0.17 (−0.55 to 0.21)
	FMV (cm^3^)	190 (42)	183 (56)	−6.7 (−24.9 to 11.6)	0.465	−0.13 (−0.49 to 0.23)
Lumbar paraspinal muscles	FF (%)	11.9 (6.9)	12.4 (6.5)	0.54 (−1.94 to 3.02)	0.662	0.08 (−0.28 to 0.44)
	TMV (cm^3^)	813 (186)	778 (191)	−35.1 (−87.7 to 17.5)	0.185	−0.18 (−0.46 to 0.09)
	FMV (cm^3^)	718 (182)	685 (185)	−33.6 (−85.5 to 18.3)	0.199	−0.18 (−0.46 to 0.1)
MILEMS to LPM FMV ratio (kg/cm^3^)		0.073 (0.030)	0.057 (0.024)	−0.016 (−0.026 to −0.005)	**0**.**006**	−0.56 (−0.98 to −0.15)
MILEMS (kg)		51.8 (23.7)	38.4 (17.2)	−13.4 (−20.6 to −6.2)	**<0**.**001**	−0.63 (−0.99 to −0.26)
LEME [time (s)]		161 (72)	64 (46)	−96.4 (−122.8 to −69.9)	**<0**.**001**	−1.57 (−2.21 to −0.94)

CI, confidence interval; CNLBP, chronic non-specific low back pain; FF, fat fraction; FMV, functional muscle volume; HV, healthy volunteers; LEME, lumbar extensor muscle endurance; LPM, lumbar paraspinal muscles; MILEMS, maximal isometric lumbar extensor muscle strength; *N*, number of subjects; TMV, total muscle volume.

Significant *P*-values are displayed in bold.

a Mean (SD).

b Paired *t*-test.

**Figure 3:**
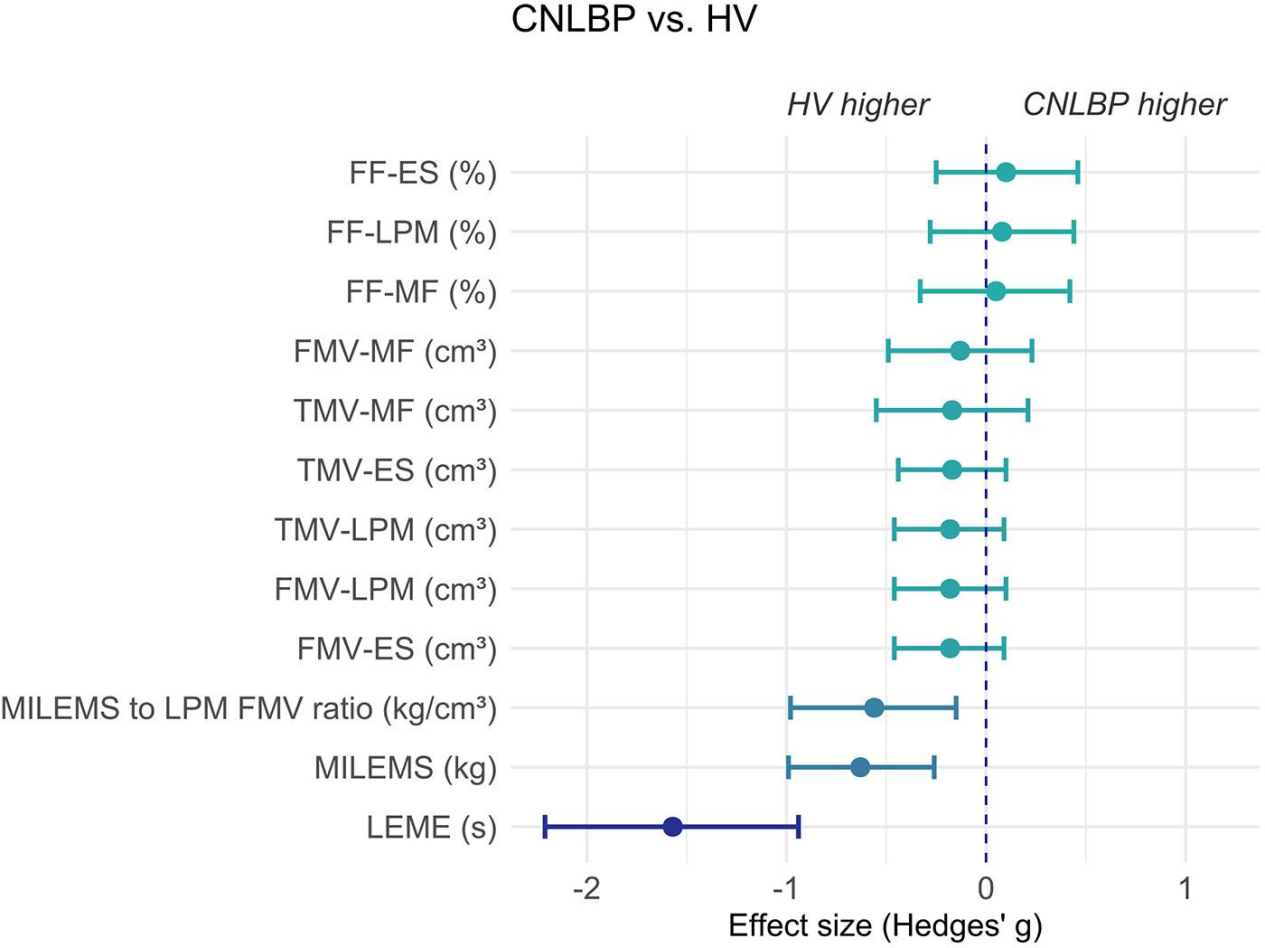
Graph showing the effect size (Hedges' *g*) and its 95% confidence interval of the difference in parameters of LPM between HV and CNLBP patients. Negative values represent results where HV evince higher values of parameters (the left side of the graph) and positive values represent results where CNLBP patients evince higher values of parameters (the right side of the graph). ES, erector spinae muscle; FF, fat fraction; FMV, functional muscle volume; LEME, lumbar extensor muscle endurance; LPM, lumbar paraspinal muscles; MF, multifidus muscle; MILEMS, maximal isometric lumbar extensor muscle strength; TMV, total muscle volume.

The groups did not differ in terms of qMRI parameters (FF, TMV, FMV) assessed for LPM as a whole, or for the multifidus and erector spinae muscles separately. In contrast, MILEMS and LEME were significantly different between groups, with CNLBP patients achieving lower values (*P* < 0.001 for all comparisons). The MILEMS to LPM FMV ratio was significantly lower in CNLBP patients (*P* = 0.006). The observed group differences in the functional parameters and the MILEMS to LPM FMV ratio, as well as the non-significant differences in the qMRI LPM parameters, were supported by the Hedges' *g* effect size estimates (Table 2, Figure 3). The difference in functional parameters between the groups was more pronounced for LEME (Hedges' *g* = −1.57, 95% confidence interval: −2.21 to −0.94, indicating a very large effect) compared to MILEMS (Hedges' *g* = −0.63, 95% confidence interval: −0.99 to −0.26, indicating a medium effect).

### 3.3 Relationship between functional and qMRI parameters of LPM in CNLBP patients

**Figure 4:**
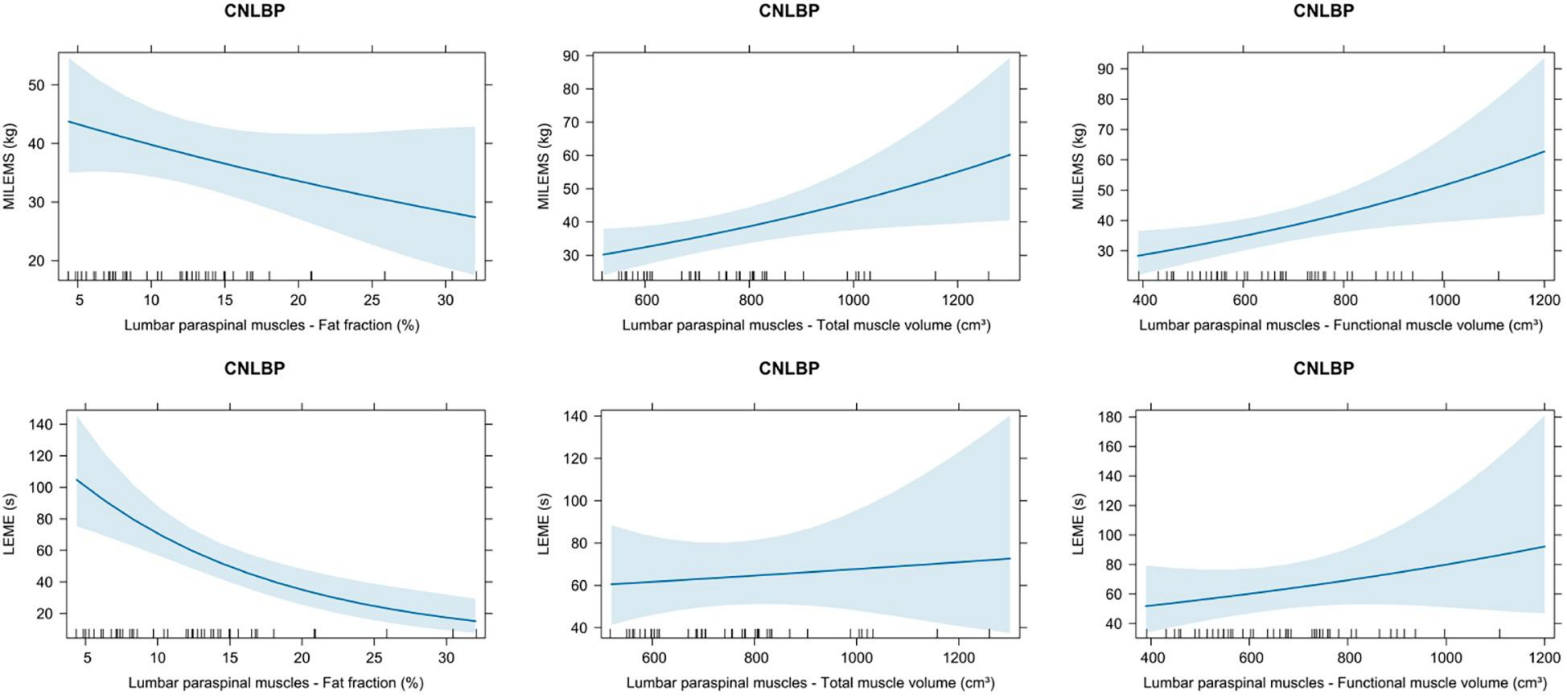
Graphs showing the relationship of quantitative MRI parameters (fat fraction, total muscle volume, functional muscle volume) and functional parameters [maximal isometric lumbar extensor muscle strength (MILEMS) and lumbar extensor muscle endurance (LEME)] of the lumbar paraspinal muscles (LPM) in patients with chronic non-specific low back pain (CNLBP). Estimated marginal means with 95% confidence intervals based on univariable generalised linear models are displayed.

We demonstrated a borderline statistically significant negative correlation between MILEMS and FF of LPM (*ρ* = −0.302; *P* = 0.049) and statistically significant positive correlations between MILEMS and both muscle volumes of LPM (*ρ* = 0.430 for TMV, *P* = 0.004; 0.468 for FMV, *P* = 0.002) (Table 3). The univariable regression models showed that each 100 cm^3^ increase in LPM TMV was associated with a statistically significant 9% increase in MILEMS, and each 100 cm^3^ increase in LPM FMV was associated with a significant 10% increase in MILEMS. However, the explained deviance of MILEMS using the TMV and FMV parameters of LPM was low (13% and 14.8%) (Table 4, Figure 4).

**Table 3:** Correlation of functional and quantitative MRI parameters of LPM in patients with CNLBP.

Parameter 1 (Functional parameter of LPM)	Parameter 2 (qMRI parameter of LPM)	Spearman coefficient	95% CI	*P*-value
MILEMS	FF	−0.302	−0.559 to 0.007	**0** **.** **049**
MILEMS	TMV	0.430	0.14 to 0.652	**0** **.** **004**
MILEMS	FMV	0.468	0.186 to 0.678	**0** **.** **002**
LEME	FF	−0.409	−0.637 to −0.115	**0** **.** **007**
LEME	TMV	0.112	−0.204 to 0.406	0.476
LEME	FMV	0.215	−0.1 to 0.491	0.166

CI, confidence interval; CNLBP, chronic non-specific low back pain; FF, fat fraction; FMV, functional muscle volume; LEME, lumbar extensor muscle endurance; LPM, lumbar paraspinal muscles; MILEMS, maximal isometric lumbar extensor muscle strength; qMRI, quantitative MRI; TMV, total muscle volume.

Significant *P*-values are displayed in bold.

**Table 4:** Univariable generalised linear regression models for LPM functional parameters in patients with CNLBP.

Predictor	Exp (Beta)	95% CI	*P*-value	Explained deviance (%)
Models for MILEMS				
(Intercept, kg)	47.1	35.1–63.5		
LPM—Fat fraction (per 10%)	0.84	0.69–1.05	0.123	5.6
(Intercept, kg)	19.1	10.5–34.4		
LPM—Total muscle volume (per 100 cm^3^)	1.09	1.02–1.18	**0** **.** **016**	13.0
(Intercept, kg)	19.4	11.3–33.3		
LPM—Functional muscle volume (per 100 cm^3^)	1.10	1.02–1.19	**0** **.** **010**	14.8
Models for LEME				
[Intercept, time (s)]	143	90.5–225		
LPM—Fat fraction (per 10%)	0.50	0.36–0.70	**<0** **.** **001**	22.1
[Intercept, time (s)]	53.6	20.3–139		
LPM—Total muscle volume (per 100 cm^3^)	1.02	0.91–1.16	0.693	0.3
[Intercept, time (s)]	39.3	15.2–100		
LPM—Functional muscle volume (per 100 cm^3^)	1.07	0.94–1.23	0.251	2.1

CI, confidence interval; CNLBP, chronic non-specific low back pain; LEME, lumbar extensor muscle endurance; LPM, lumbar paraspinal muscles; MILEMS, maximal isometric lumbar extensor muscle strength.

Significant *P*-values are displayed in bold.

For LEME, the only statistically significant correlation was found with FF of LPM (*ρ* = −0.409; *P* = 0.007) (Table 3). According to the regression model, every 10% increase in the FF was associated with a 50% decrease in LEME (*P* < 0.001), but the explained deviance was a modest 22.1% (Table 4, Figure 4). There was no significant correlation between LEME and LPM volumes.

### 3.4 Effect of exercise therapy on functional and qMRI parameters of LPM in CNLBP patients

We compared LPM parameters before and after exercise therapy in 27 CNLBP patients and there was no significant change in any muscle qMRI parameter (FF, TMV, or FMV, both LPM as a whole, and erector spinae muscle or multifidus muscle separately) (Table 5). On the other hand, the functional parameters of the LPM (MILEMS and LEME), the MILEMS to LPM FMV ratio, and all types of pain and disability improved significantly with exercise (each *P* < 0.001).

**Table 5:** Quantitative MRI, functional parameters of LPM, and patient-oriented outcomes before and after exercise in CNLBP patients.

Parameter		CNLBP (before exercise therapy) *N* = 27^a^	CNLBP (after exercise therapy) *N* = 27^a^	Mean difference (95% CI)	*P*-value^b^	Hedges' *g* (95% CI)
Erector spinae muscle	FF (%)	12.0 (6.5)	11.7 (6.2)	−0.32 (−1.16 to 0.51)	0.433	−0.05 (−0.17 to 0.07)
	TMV (cm^3^)	555 (150)	552 (156)	−3.4 (−17.8 to 11.0)	0.632	−0.02 (−0.11 to 0.07)
	FMV (cm^3^)	489 (141)	490 (151)	0.5 (−10.0 to 11.1)	0.915	0 (−0.06 to 0.07)
Multifidus muscle	FF (%)	16.1 (10.2)	15.9 (10.3)	−0.24 (−1.26 to 0.77)	0.626	−0.02 (−0.12 to 0.07)
	TMV (cm^3^)	216 (60)	213 (61)	−3.2 (−11.0 to 4.6)	0.412	−0.05 (−0.17 to 0.07)
	FMV (cm^3^)	183 (59)	182 (63)	−1.4 (−6.9 to 4.1)	0.617	−0.02 (−0.1 to 0.06)
Lumbar paraspinal muscles	FF (%)	13.2 (7.3)	12.9 (7.2)	−0.32 (−1.14 to 0.50)	0.427	−0.04 (−0.15 to 0.06)
	TMV (cm^3^)	771 (197)	765 (205)	−6.6 (−26.1 to 13.0)	0.497	−0.03 (−0.12 to 0.06)
	FMV (cm^3^)	672 (191)	672 (205)	−0.8 (−14.6 to 13.0)	0.905	0 (−0.06 to 0.06)
MILEMS to LPM FMV ratio (kg/cm^3^)		0.059 (0.027)	0.084 (0.030)	0.025 (0.018 to 0.031)	**<0**.**001**	0.83 (0.58 to 1.07)
MILEMS (kg)		39 (18)	55 (21)	16.1 (11.5 to 20.6)	**<0**.**001**	0.79 (0.54 to 1.04)
LEME [time (s)]		66 (50)	126 (54)	60.6 (44.2 to 76.9)	**<0**.**001**	1.12 (0.75 to 1.5)
Pain intensity (NRS 0–10)	Average	4.0 (1.9)	2.0 (1.2)	−2.0 (−2.5 to −1.5)	**<0** **.** **001**	−1.06 (−1.38 to −0.74)
	Current	2.6 (1.9)	1.2 (1.4)	−1.4 (−2.1 to −0.8)	**<0** **.** **001**	−0.8 (−1.18 to −0.42)
	Maximum	6.2 (2.3)	3.4 (1.6)	−2.8 (−3.8 to −1.8)	**<0** **.** **001**	−1.36 (−2 to −0.71)
Oswestry Disability Index (0%–100%)		17.7 (7.9)	4.6 (4.4)	−13.1 (−15.8 to −10.3)	**<0**.**001**	−1.86 (−2.49 to −1.23)
Roland-Morris Disability Questionnaire (0–24)		7.9 (4.6)	1.9 (1.9)	−6.0 (−7.7 to −4.4)	**<0**.**001**	−1.52 (−2.1 to −0.93)

CI, confidence interval; CNLBP, chronic non-specific low back pain; FF, fat fraction; FMV, functional muscle volume; LEME, lumbar extensor muscle endurance; LPM, lumbar paraspinal muscles; MILEMS, maximal isometric lumbar extensor muscle strength; *N*, number of subjects; NRS, numerical rating scale; TMV, total muscle volume.

Significant *P*-values are displayed in bold.

a Mean (SD).

b Paired *t*-test.

**Figure 5:**
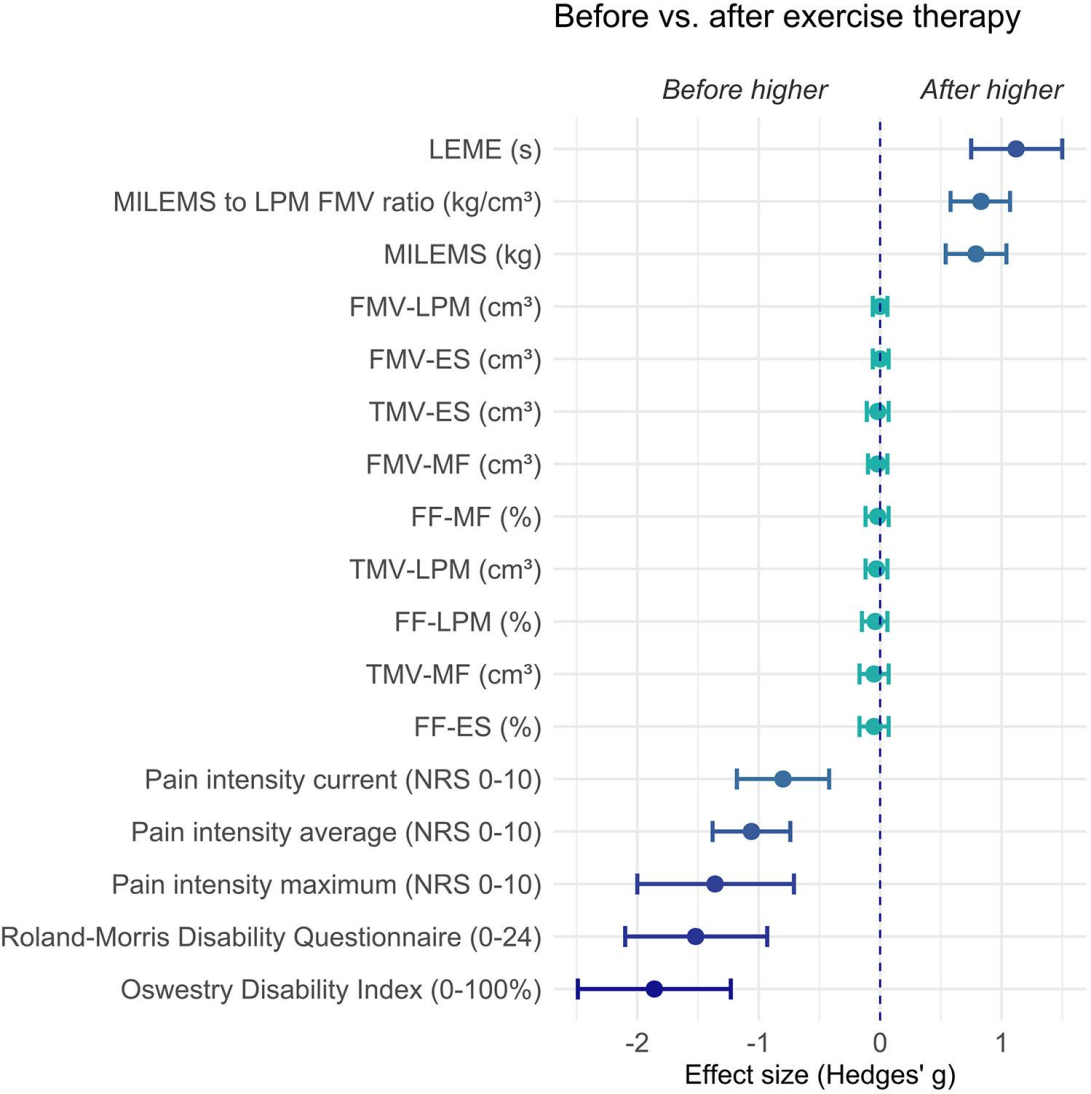
Graph showing the effect size (Hedges' *g*) and its 95% confidence interval of the difference in parameters in CNLBP patients before versus after exercise therapy. Negative values represent results where CNLBP patients evince higher values before exercise therapy (the left side of the graph) and positive values represent results where CNLBP patients evince higher values after exercise therapy (the right side of the graph). ES, erector spinae muscle; FF, fat fraction; FMV, functional muscle volume; LEME, lumbar extensor muscle endurance; LPM, lumbar paraspinal muscles; MF, multifidus muscle; MILEMS, maximal isometric lumbar extensor muscle strength; NRS, numerical rating scale; TMV, total muscle volume.

The effect size also confirmed a marked change after exercise therapy in functional parameters of LPM (Hedges' *g* = 0.79, 95% confidence interval 0.54 to 1.04 for MILEMS and Hedges' *g* = 1.12, 95% confidence interval 0.75 to 1.5 for LEME) and disability and pain (Hedges' *g* ranging from −0.8 to −1.86) including the MILEMS to LPM FMV ratio (Hedges' *g* = 0.83, 95% confidence interval 0.58 to 1.07) (Table 5, Figure 5).

**Figure 6:**
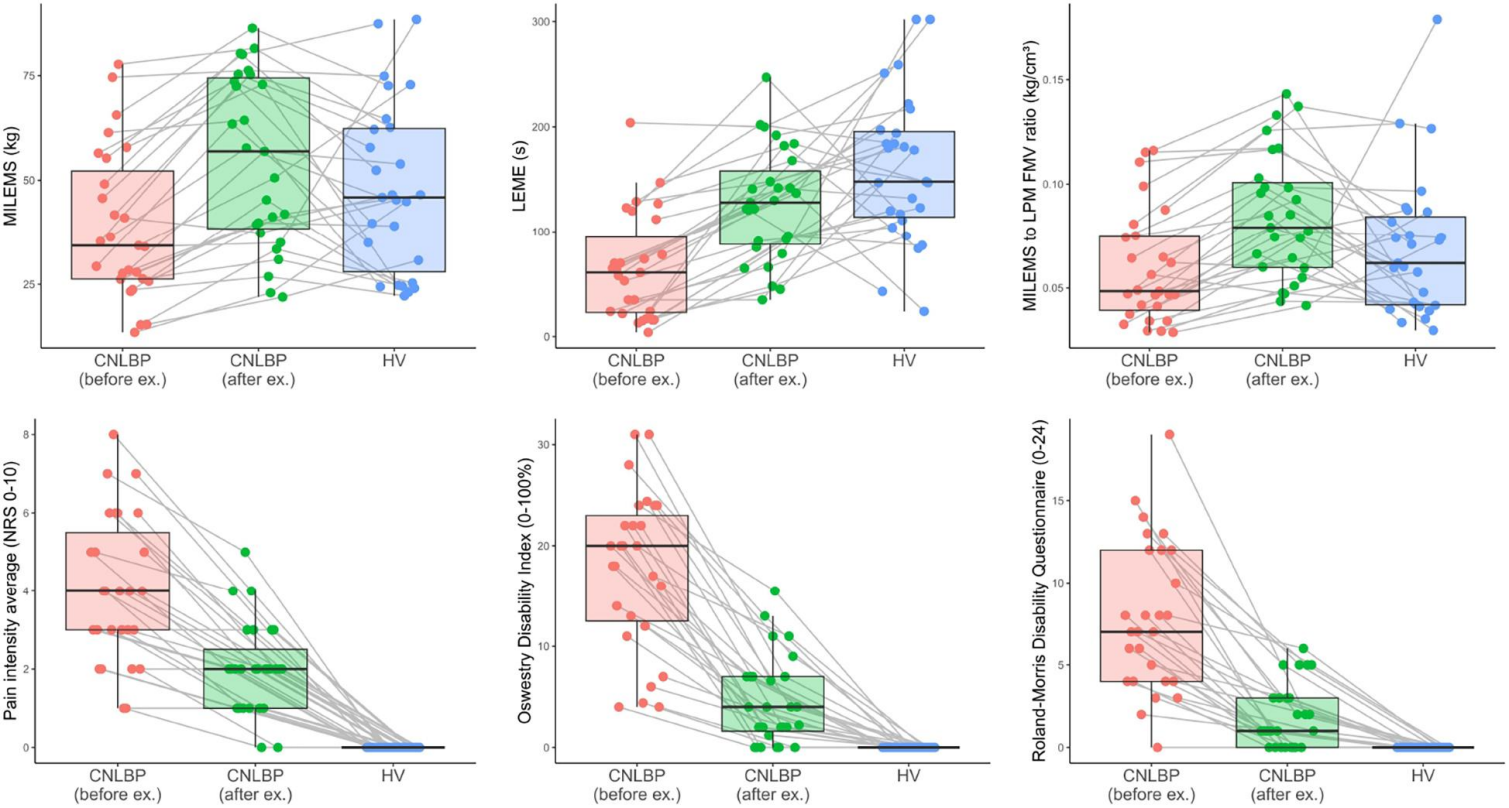
Box plots showing a comparison of the selected parameters for CNLBP patients before and after exercise therapy, and for HV; the bold line represents the median, the box boundaries correspond to the 25th and 75th percentiles, and the whiskers indicate non-outlier observed values. Each observation is represented as a single dot. The values of each patient before and after exercise therapy and the matched HV are connected by a grey line. FMV, functional muscle volume; LEME, lumbar extensor muscle endurance; LPM, lumbar paraspinal muscles; MILEMS, maximal isometric lumbar extensor muscle strength; NRS, numerical rating scale.

The post-exercise values of MILEMS and MILEMS to LPM FMV ratio in CNLBP patients were no longer significantly different from matched HV. LEME after exercise was borderline statistically significantly lower in CNLBP patients compared to HV (*P* = 0.048). However, disability and pain were consistently higher in CNLBP patients even after exercise compared with matched HV (Table 6, Figure 6).

**Table 6:** Comparison of improved post-exercise parameters in CNLBP patients with matched HV.

Parameter		HV *N* = 27^a^	CNLBP (after exercise therapy) *N* = 27^a^	Mean difference (95% CI)	*P*-value^b^	Hedges' *g* (95% CI)
MILEMS to LPM FMV ratio (kg/cm^3^)		0.069 (0.034)	0.084 (0.030)	0.015 (−0.001 to 0.031)	0.069	0.44 (−0.05 to 0.93)
MILEMS (kg)		48 (20)	55 (21)	7.1 (−1.2 to 15.4)	0.091	0.34 (−0.06 to 0.73)
LEME [time (s)]		161 (71)	126 (54)	−34.2 (−68.0 to −0.4)	**0** **.** **048**	−0.53 (−1.07 to 0.02)
Pain intensity (NRS 0–10)	Average	0.0 (0.0)	2.0 (1.2)	2.0 (1.6–2.5)	**<0**.**001**	2.41 (1.36–3.46)
	Current	0.0 (0.0)	1.2 (1.4)	1.2 (0.6–1.7)	**<0**.**001**	1.17 (0.48–1.86)
	Maximum	0.0 (0.0)	3.4 (1.6)	3.4 (2.8–4.0)	**<0**.**001**	2.92 (1.71–4.14)
Oswestry Disability Index (0%–100%)		0.0 (0.0)	4.6 (4.4)	4.6 (2.8–6.3)	**<0** **.** **001**	1.44 (0.68–2.19)
Roland-Morris Disability Questionnaire (0–24)		0.0 (0.0)	1.9 (1.9)	1.9 (1.1–2.6)	**<0** **.** **001**	1.36 (0.62–2.09)

CI, confidence interval; CNLBP, chronic non-specific low back pain; FMV, functional muscle volume; HV, healthy volunteers; LEME, lumbar extensor muscle endurance; LPM, lumbar paraspinal muscles; MILEMS, maximal isometric lumbar extensor muscle strength; *N*, number of subjects; NRS, numerical rating scale.

Significant *P*-values are displayed in bold.

a Mean (SD).

b Paired *t*-test.

## 4 Discussion

The present study investigated functional and qMRI parameters of the LPM in CNLBP patients, their interrelationship, and the effect of home-based exercise therapy on these parameters. CNLBP patients showed significant LPM dysfunction (reduced strength and endurance) and impaired LPM quality compared to matched HV, although the two groups did not differ significantly in LPM qMRI parameters (FF, muscle volumes). A correlation between the qMRI parameters of LPM and MILEMS was demonstrated, stronger for LPM volume parameters than for FF, as was a correlation between LEME and LPM FF; however, correlation coefficients and variability of MILEMS and LEME explained by qMRI parameters were relatively low. After 18-week home-based exercise therapy, LPM functional parameters improved significantly, as did patient-oriented outcomes (reduction in pain and disability). The qMRI parameters of the LPM remained the same after exercise, however MILEMS to LPM FMV ratio showed a clear improvement, indicating an improvement in LPM muscle quality. Therefore, our study does not support the use of qMRI parameters as objective and reliable biomarkers of LPM dysfunction in a cohort of CNLBP with mild clinical impairment.

The evidence of LPM deconditioning (decrease in muscle strength and endurance) in CNLBP patients in the present study supports the findings of previously published studies (9, 18, 39, 40). However, the effect sizes of differences between individuals with and without CNLBP in those studies varied (18). In this study, the effect size of differences in functional parameters based on Hedges' *g* between HV and CNLBP was more pronounced for endurance than for strength of LPM. This suggests that LPM endurance is more affected than strength in CNLBP patients.

Many studies have demonstrated the presence of atrophy (17, 41, 42), fat infiltration (17, 43–45), and connective tissue accumulation in the back muscles, particularly in the deep fibres of the multifidus muscle in CNLBP patients (9). There is no consensus whether morphological changes affect the multifidus muscle and erector spinae or the multifidus muscle only (18). However, other studies found no significant association between some of the LPM morphological changes and LBP (46, 47). The present study showed no difference between CNLBP patients and HV in qMRI parameters (FF, volumes) of the LPM as a whole or separately in erector spinae muscle and multifidus muscle. A study by Wesselink et al. (17) reported higher mean FF values than our study in participants with no low back pain (36.2% vs. 14.3% for multifidus muscle, 27.0% vs. 10.8% for erector spinae muscle). Wesselink et al. also reported significantly higher mean FF values in the multifidus and erector spinae muscles in patients with chronic low back pain than in people with no pain, with a mean difference ≤1.6%. These discrepancies are likely due to several factors. First, we utilised 6-point gradient echo Dixon sequences; Wesselink et al. used 2-point turbo spin echo Dixon sequences, which are more biased by T2 decay and are not corrected for different fat peaks. An evaluation of different Dixon sequences showed that variations in FF values can reach up to 33% when comparing 6-point and 2-point gradient echo Dixon sequences (48). In another article, Grimm et al. (49) showed high correlation and reproducibility of 6-point gradient echo Dixon sequences with MR spectroscopy, which enhances the credibility of our results. Our study sample was also considerably smaller than that of Wesselink et al. (43 matched pairs vs. 6,953 subjects with no back pain and 1,681 subjects with chronic low back pain), which could have affected the statistical significance of the difference. On the other hand, a thorough neurological examination was performed and the intensity and severity of CNLBP was assessed in each subject in our study; this assessment was not available in the large cohort from UK Biobank used in the Wesselink et al. study. The severity and duration of LBP may also play a role. Our study group comprised patients with milder back pain; longer and more severe complaints (greater disability) have been associated with more pronounced muscle morphological changes (9, 17, 18, 50). Also, paraspinal muscles can be affected by denervation that leads to morphological changes in patients with chronic LBP and root compression (51); these patients were excluded from our study.

Importantly, the MILEMS to LPM FMV ratio, a parameter reflecting the quality of the muscle by expressing the strength generated by 1 cm^3^ of LPM muscle mass (fat free) (35), was significantly lower in CNLBP patients and increased significantly after exercise therapy. Muscle function depends on muscle quality, which is influenced by factors including muscle fibre type, muscle architecture, metabolism, aerobic capacity, insulin resistance, fat infiltration, fibrosis, and neuromuscular activation (35, 52). In addition, the change in muscle quality may precede loss of muscle mass (52), which is in concordance with our results where we did not observe a difference in muscle volume, but did demonstrate a difference in LPM quality and function in CNLBP patients.

Although strength of LPM decreased with increasing LPM FF and decreasing volumes, and endurance decreased with increasing FF, correlation coefficients and coefficients of determination were relatively low. Similarly, in HV and patients with myotonic dystrophy type 2, the correlation between functional and qMRI parameters of LPM was not strong (7, 8). This poor correlation could reside in multiple factors. Firstly, intrinsic motivation and psychosocial factors affect physical performance (53–55). Furthermore, MILEMS and LEME are not the result of isolated LPM or LEM activity; they can be influenced by the involvement of other muscle groups. Additionally, muscle mass is also replaced by non-contractile tissues, such as fibrosis, which is included in FMV. Thus correlations between qMRI parameters and muscle function can be affected. Other MRI methods could be beneficial, namely methods using ultrashort echo times or magnetic resonance elastography to image fibrosis and diffusion tensor imaging of LPM for the identification of muscle microstructure (56, 57).

For patients with chronic LBP, sensorimotor control exercises that focus on reducing the overactivation of the superficial erector spinae musle while improving the function of the deeper muscles (including multifidus muscle) are recommended (18). These exercises were also used in the current study, significantly affecting MILEMS and LEME, apparently due to an improvement in muscle quality (contractility) and positive changes in the recruitment and coordination in the back muscles, expected after this kind of exercise (18). The functional changes were accompanied by improved subjective perception of pain and disability, consistent with findings described in another study (58). However, no change in qMRI parameters of LPM was observed in our study. Thus, the improvement in clinical parameters in CNLBP patients after exercise observed in our study can be primarily attributed to the mechanisms of neuromuscular reactivation and sensorimotor reorganisation. A recent systematic review concluded that paraspinal fatty infiltration is not reversible through exercise in people with LBP (6). Conversely, some studies report an increase in lower trunk muscle size following an exercise programme; however, this effect is highly dependent on the type and duration of exercise (18, 59). Further research is needed to determine the type, duration and intensity of exercise capable of inducing structural changes in LPM.

This study has limitations. Due to the single-centre study and the strict eligibility criteria, the number of CNLBP patients was limited. A power analysis estimated a total of 47 patients as the sample size. Even though 50 patients were screened, only 43 were willing to follow the whole measurement protocol. Due to the Covid-19 pandemic, we were not able to recruit more subjects. Patients with lower average pain and disability predominated, which may have influenced the measurements. Therefore, the results can be applied only to CNLBP patients with a milder degree of clinical impairment. Also, the methodology of functional examination has its shortcomings, as MILEMS and LEME are not the result of isolated LPM or LEM activity; the lack of blinded assessors could have introduced potential bias; the follow-up period of 18 weeks was relatively short; and structural adaptations in the studied muscles might have required longer to evolve. Additionally, we evaluated LPM FF and volumes; however, degenerative changes in muscles are also accompanied by replacement by non-contractile tissue, such as fibrosis, and by changes in muscle fibre orientation. Future studies should therefore implement multimodal MRI protocols utilising diffusion-weighted imaging metrics, ultrashort echo times, or MR elastography. Finally, the absence of a control group for the exercise intervention limits the causal inference. On the other hand, the study benefits from the homogeneity of the CNLBP patient cohort (a thorough clinical and MRI examination was aimed at excluding pain other than non-specific LBP), the comparison with matched HV, and a very detailed functional and qMRI examination of the LPM, including the assessment of qMRI parameters of LPM across the entire lumbar spine.

This study contributed to the understanding of LPM involvement in CNLBP. It also highlighted the improvement in dysfunction and muscle quality of the LPM with exercise therapy, accompanied by clinical improvement. In patients with milder clinical impairment, functional testing of the LPM appears to be more beneficial than MRI of these muscles, but both examinations should be considered complementary and neither should be ruled out. The results of this study need to be further validated by additional prospective studies to understand the importance of LPM dysfunction in the development of CNLBP and by blinded controlled studies to assess the effect of exercise on LPM function and clinical status in CNLBP patients.

## 5 Conclusion

This study confirmed the presence of significant dysfunction and impaired quality of LPM in CNLBP patients; this almost completely improved after home-based exercise therapy to the level of HV and was accompanied by clinical improvement.

Our study does not support the use of qMRI parameters as objective and reliable biomarkers of LPM dysfunction in a cohort of CNLBP with mild clinical impairment because there was no strong correlation between LPM function and morphology, and no significant difference in qMRI between CNLBP and matched HV groups, nor between CNLBP patients before and after the exercise programme.

## Data Availability

The raw data supporting the conclusions of this article will be made available by the authors, without undue reservation.

## References

[1] Balagué F Mannion AF Pellisé F Cedraschi C. Non-specific low back pain. Lancet. (2012) 379:482–91. 10.1016/S0140-6736(11)60610-7 21982256

[2] Nicol V Verdaguer C Daste C Bisseriex H Lapeyre É Lefèvre-Colau M-M Chronic low back pain: a narrative review of recent international guidelines for diagnosis and conservative treatment. J Clin Med. (2023) 12:1685. 10.3390/jcm12041685 36836220 PMC9964474

[3] Maher C Underwood M Buchbinder R. Non-specific low back pain. Lancet. (2017) 389:736–47. 10.1016/S0140-6736(16)30970-9 27745712

[4] Airaksinen O Brox JI Cedraschi C Hildebrandt J Klaber-Moffett J Kovacs F Chapter 4. European guidelines for the management of chronic nonspecific low back pain. Eur Spine J. (2006) 15(Suppl 2):S192–300. 10.1007/s00586-006-1072-1 16550448 PMC3454542

[5] O’Sullivan P Caneiro JP O’Keeffe M O’Sullivan K. Unraveling the complexity of low back pain. J Orthop Sports Phys Ther. (2016) 46:932–7. 10.2519/jospt.2016.0609 27802794

[6] Wesselink EO Pool JJM Mollema J Weber KA Elliott JM Coppieters MW Is fatty infiltration in paraspinal muscles reversible with exercise in people with low back pain? A systematic review. Eur Spine J. (2023) 32:787–96. 10.1007/s00586-022-07471-w 36459201 PMC10515728

[7] Kokosova V Krkoska P Vlazna D Sladeckova M Dostal M Kerkovsky M Quantitative magnetic resonance imaging parameters of lumbar paraspinal muscle impairment in myotonic dystrophy type 2 and their evolution with aging. Front Neurol. (2025) 16:1525952. 10.3389/fneur.2025.1525952 40046670 PMC11879826

[8] Kokosova V Krkoska P Vlazna D Dostal M Ovesna P Matulov K Quantitative magnetic resonance imaging parameters of lumbar paraspinal muscles and their relationship to function and ageing in healthy subjects. Quant Imaging Med Surg. (2025) 15:6517–25. 10.21037/qims-2024-2633 40727326 PMC12290670

[9] Hodges PW Danneels L. Changes in structure and function of the back muscles in low back pain: different time points, observations, and mechanisms. J Orthop Sports Phys Ther. (2019) 49:464–76. 10.2519/jospt.2019.8827 31151377

[10] Dahlqvist JR Vissing CR Hedermann G Thomsen C Vissing J. Fat replacement of paraspinal muscles with aging in healthy adults. Med Sci Sports Exerc. (2017) 49:595–601. 10.1249/MSS.0000000000001119 27741218

[11] Schlaeger S Inhuber S Rohrmeier A Dieckmeyer M Freitag F Klupp E Association of paraspinal muscle water-fat MRI-based measurements with isometric strength measurements. Eur Radiol. (2019) 29:599–608. 10.1007/s00330-018-5631-8 30014202

[12] Burian E Inhuber S Schlaeger S Dieckmeyer M Klupp E Franz D Association of thigh and paraspinal muscle composition in young adults using chemical shift encoding-based water–fat MRI. Quant Imaging Med Surg. (2020) 10:128–36. 10.21037/qims.2019.11.08 31956536 PMC6960431

[13] Crawford RJ Filli L Elliott JM Nanz D Fischer MA Marcon M Age- and level-dependence of fatty infiltration in lumbar paravertebral muscles of healthy volunteers. AJNR Am J Neuroradiol. (2016) 37:742–8. 10.3174/ajnr.A4596 26635285 PMC7960169

[14] Huang R Pan F Kong C Lu S. Age- and sex-dependent differences in the morphology and composition of paraspinal muscles between subjects with and without lumbar degenerative diseases. BMC Musculoskelet Disord. (2022) 23:734. 10.1186/s12891-022-05692-0 35915426 PMC9341069

[15] Krkoska P Kokosova V Dostal M Vlazna D Kerkovsky M Straka M Assessment of lumbar paraspinal muscle morphology using mDixon quant magnetic resonance imaging (MRI): a cross-sectional study in healthy subjects. Quant Imaging Med Surg. (2024) 14:6015–35. 10.21037/qims-23-1796 39144006 PMC11320528

[16] Vlazna D Adamova B Krkoska P Kokosova V Matulova K Barusova T Strength and endurance of the lumbar extensor muscles and their predictors: a cross-sectional study in healthy subjects. J Electromyogr Kinesiol. (2025) 80:102973. 10.1016/j.jelekin.2024.102973 39765107

[17] Wesselink EO Pool-Goudzwaard A De Leener B Law CSW Fenyo MB Ello GM Investigating the associations between lumbar paraspinal muscle health and age, BMI, sex, physical activity, and back pain using an automated computer-vision model: a UK biobank study. Spine. (2024) 24:1253–66. 10.1016/j.spinee.2024.02.013 PMC11779699 38417587

[18] Matheve T Hodges P Danneels L. The role of back muscle dysfunctions in chronic low back pain: state-of-the-art and clinical implications. J Clin Med. (2023) 12:5510. 10.3390/jcm12175510 37685576 PMC10487902

[19] Rosenstein B Montpetit C Vaillancourt N Dover G Weiss C Papula LA Aquatic exercise versus standard care on paraspinal muscle morphology and function in chronic low back pain patients: a randomized controlled trial. Sci Rep. (2025) 15:15798. 10.1038/s41598-025-00210-3 40328824 PMC12056110

[20] Rosenstein B Rye M Roussac A Naghdi N Macedo LG Elliott J Comparison of combined motor control training and isolated extensor strengthening versus general exercise on lumbar paraspinal muscle health and associations with patient-reported outcome measures in chronic low back pain patients: a randomized controlled trial. Glob Spine J. (2025) 15:3332–49. 10.1177/21925682251324490 PMC11897994 40066720

[21] Corp N Mansell G Stynes S Wynne-Jones G Morsø L Hill JC Evidence-based treatment recommendations for neck and low back pain across Europe: a systematic review of guidelines. Eur J Pain Lond Engl. (2021) 25:275–95. 10.1002/ejp.1679 PMC7839780 33064878

[22] Shibukawa S Yoshimaru D Hiyama Y Saho T Ozawa T Usui K Predicting perceived exertion during high-intensity exercise using quantitative MRI: insights from T2* value and muscle cross-sectional area. Radiol Phys Technol. (2025) 18:746–55. 10.1007/s12194-025-00927-w 40518471

[23] Sorrentino RG Vovk A Šuput D Ioannou LG Mekjavic V Fernandez-Gonzalo R Enhancement of muscle activation during squat exercise: evaluation with magnetic resonance imaging. Eur J Appl Physiol. (2025). 10.1007/s00421-025-05856-5 40593201 PMC12678469

[24] Botta RM Palermi S Tarantino D. High-intensity interval training for chronic pain conditions: a narrative review. J Exerc Rehabil. (2022) 18:10–9. 10.12965/jer.2142718.359 35356137 PMC8934613

[25] Meus T Timmermans A Klaps S Verbrugghe J. High-intensity training telerehabilitation for persons with chronic low back pain: a pilot clinical trial. J Clin Med. (2024) 13:7599. 10.3390/jcm13247599 39768521 PMC11676959

[26] Dosbaba F Senkyr V Vlazna D Minarikova J Nevelikova M Sladeckova M Comparison of hybrid guided home-based and outpatient rehabilitation in patients with chronic low back pain: a randomized controlled trial. J Bodyw Mov Ther. (2025) 45:110–8. 10.1016/j.jbmt.2025.08.009 41316561

[27] Lentz TA Coffman CJ Cope T Stearns Z Simon CB Choate A If you build it, will they come? Patient and provider use of a novel hybrid telehealth care pathway for low back pain. Phys Ther. (2024) 104:pzad127. 10.1093/ptj/pzad127 37756618 PMC10851867

[28] Fairbank JC Couper J Davies JB O’Brien JP. The oswestry low back pain disability questionnaire. Physiotherapy. (1980) 66:271–3. 6450426

[29] Roland M Fairbank J. The roland–morris disability questionnaire and the oswestry disability questionnaire. Spine. (2000) 25:3115. 10.1097/00007632-200012150-00006 11124727

[30] Vlažná D Krkoška P Kuhn M Dosbaba F Batalik L Vlčková E Assessment of lumbar extensor muscles in the context of trunk function, a pilot study in healthy individuals. Appl Sci. (2021) 11:9518. 10.3390/app11209518

[31] Yushkevich PA Piven J Hazlett HC Smith RG Ho S Gee JC User-guided 3D active contour segmentation of anatomical structures: significantly improved efficiency and reliability. NeuroImage. (2006) 31:1116–28. 10.1016/j.neuroimage.2006.01.015 16545965

[32] Carlier PG Marty B Scheidegger O Loureiro de Sousa P Baudin P-Y Snezhko E Skeletal muscle quantitative nuclear magnetic resonance imaging and spectroscopy as an outcome measure for clinical trials. J Neuromuscul Dis. (2016) 3:1–28. 10.3233/JND-160145 27854210 PMC5271435

[33] Dahlqvist JR Oestergaard ST Poulsen NS Knak KL Thomsen C Vissing J. Muscle contractility in spinobulbar muscular atrophy. Sci Rep. (2019) 9:4680. 10.1038/s41598-019-41240-y 30886222 PMC6423126

[34] Jones EJ Bishop PA Woods AK Green JM. Cross-sectional area and muscular strength: a brief review. Sports Med. (2008) 38:987–94. 10.2165/00007256-200838120-00003 19026016

[35] Kuschel LB Sonnenburg D Engel T. Factors of muscle quality and determinants of muscle strength: a systematic literature review. Healthcare. (2022) 10:1937. 10.3390/healthcare10101937 36292384 PMC9601777

[36] Krkoska P Vlazna D Sladeckova M Minarikova J Barusova T Batalik L Adherence and effect of home-based rehabilitation with telemonitoring support in patients with chronic non-specific low back pain: a pilot study. Int J Environ Res Public Health. (2023) 20:1504. 10.3390/ijerph20021504 36674258 PMC9860722

[37] Hedges VL Olkin I. Statistical Methods for Meta-Analysis. Cambridge: Academic Press (1985). 10.1016/C2009-0-03396-0

[38] Sawilowsky SS. New effect size rules of thumb. J Mod Appl Stat Methods. (2009) 8:597–9. 10.56801/10.56801/v8.i.452

[39] Conway R Behennah J Fisher J Osborne N Steele J. A comparison of isolated lumbar extension strength between healthy asymptomatic participants and chronic low back pain participants without previous lumbar spine surgery. Spine. (2018) 43:E1232–7. 10.1097/BRS.0000000000002701 29689006

[40] Steele J Bruce-Low S Smith D. A reappraisal of the deconditioning hypothesis in low back pain: review of evidence from a triumvirate of research methods on specific lumbar extensor deconditioning. Curr Med Res Opin. (2014) 30:865–911. 10.1185/03007995.2013.875465 24328452

[41] Fortin M Macedo LG. Multifidus and paraspinal muscle group cross-sectional areas of patients with low back pain and control patients: a systematic review with a focus on blinding. Phys Ther. (2013) 93:873–88. 10.2522/ptj.20120457 23504343 PMC3704232

[42] Kader DF Wardlaw D Smith FW. Correlation between the MRI changes in the lumbar multifidus muscles and leg pain. Clin Radiol. (2000) 55:145–9. 10.1053/crad.1999.0340 10657162

[43] Kjaer P Bendix T Sorensen JS Korsholm L Leboeuf-Yde C. Are MRI-defined fat infiltrations in the multifidus muscles associated with low back pain? BMC Med. (2007) 5:2. 10.1186/1741-7015-5-2 17254322 PMC1796893

[44] Mengiardi B Schmid MR Boos N Pfirrmann CWA Brunner F Elfering A Fat content of lumbar paraspinal muscles in patients with chronic low back pain and in asymptomatic volunteers: quantification with MR spectroscopy. Radiology. (2006) 240:786–92. 10.1148/radiol.2403050820 16926328

[45] Seyedhoseinpoor T Taghipour M Dadgoo M Sanjari MA Takamjani IE Kazemnejad A Alteration of lumbar muscle morphology and composition in relation to low back pain: a systematic review and meta-analysis. Spine J. (2022) 22:660–76. 10.1016/j.spinee.2021.10.018 34718177

[46] Danneels LA Vanderstraeten GG Cambier DC Witvrouw EE De Cuyper HJ Danneels L. CT imaging of trunk muscles in chronic low back pain patients and healthy control subjects. Eur Spine J. (2000) 9:266–72. 10.1007/s005860000190 11261613 PMC3611341

[47] Kalichman L Hodges P Li L Guermazi A Hunter DJ. Changes in paraspinal muscles and their association with low back pain and spinal degeneration: CT study. Eur Spine J. (2010) 19:1136–44. 10.1007/s00586-009-1257-5 20033739 PMC2900015

[48] Grimm A Meyer H Nickel MD Nittka M Raithel E Chaudry O Evaluation of 2-point, 3-point, and 6-point Dixon magnetic resonance imaging with flexible echo timing for muscle fat quantification. Eur J Radiol. (2018) 103:57–64. 10.1016/j.ejrad.2018.04.011 29803386

[49] Grimm A Meyer H Nickel MD Nittka M Raithel E Chaudry O Repeatability of Dixon magnetic resonance imaging and magnetic resonance spectroscopy for quantitative muscle fat assessments in the thigh. J Cachexia Sarcopenia Muscle. (2018) 9:1093–100. 10.1002/jcsm.12343 30221479 PMC6240750

[50] Goubert D Oosterwijck JV Meeus M Danneels L. Structural changes of lumbar muscles in non-specific low back pain: a systematic review. Pain Physician. (2016) 19:E985–1000. 27676689

[51] Yoshihara K Nakayama Y Fujii N Aoki T Ito H. Atrophy of the multifidus muscle in patients with lumbar disk herniation: histochemical and electromyographic study. Orthopedics. (2003) 26:493–5. 10.3928/0147-7447-20030501-14 12755213

[52] McGregor RA Cameron-Smith D Poppitt SD. It is not just muscle mass: a review of muscle quality, composition and metabolism during ageing as determinants of muscle function and mobility in later life. Longev Heal. (2014) 3:9. 10.1186/2046-2395-3-9 PMC4268803 25520782

[53] Mannion AF O’Riordan D Dvorak J Masharawi Y. The relationship between psychological factors and performance on the biering-sørensen back muscle endurance test. Spine J. (2011) 11:849–57. 10.1016/j.spinee.2011.08.004 21903483

[54] Matheve T Janssens L Goossens N Danneels L Willems T Van Oosterwijck J The relationship between pain-related psychological factors and maximal physical performance in low back pain: a systematic review and meta-analysis. J Pain. (2022) 23:2036–51. 10.1016/j.jpain.2022.08.001 36057387

[55] Smith AJ O’Sullivan PB Campbell AC Straker LM. The relationship between back muscle endurance and physical, lifestyle, and psychological factors in adolescents. J Orthop Sports Phys Ther. (2010) 40:517–23. 10.2519/jospt.2010.3369 20508326

[56] Klupp E Cervantes B Schlaeger S Inhuber S Kreuzpointer F Schwirtz A Paraspinal muscle DTI metrics predict muscle strength. J Magn Reson Imaging. (2019) 50:816–23. 10.1002/jmri.26679 30723976 PMC6767405

[57] Lenchik L Mazzoli V Cawthon PM Hepple RT Boutin RD. Muscle steatosis and fibrosis in older adults, from the AJR special series on imaging of fibrosis. Am J Roentgenol. (2024) 222:e2329742. 10.2214/AJR.23.29742 37610777

[58] Steele J Fisher J Perrin C Conway R Bruce-Low S Smith D. Does change in isolated lumbar extensor muscle function correlate with good clinical outcome? A secondary analysis of data on change in isolated lumbar extension strength, pain, and disability in chronic low back pain. Disabil Rehabil. (2019) 41:1287–95. 10.1080/09638288.2018.1424952 29327605

[59] Shahtahmassebi B Hebert JJ Stomski NJ Hecimovich M Fairchild TJ. The effect of exercise training on lower trunk muscle morphology. Sports Med. (2014) 44:1439–58. 10.1007/s40279-014-0213-7 25015476

